# DNA Damage Protecting Activity and Free Radical Scavenging Activity of Anthocyanins from Red Sorghum (*Sorghum bicolor*) Bran

**DOI:** 10.1155/2012/258787

**Published:** 2012-02-08

**Authors:** P. Suganya Devi, M. Saravana Kumar, S. Mohan Das

**Affiliations:** ^1^P.G. Department of Biotechnology, Dr. Mahalingam Centre for Research and Development, N.G.M. College, Pollachi 642001, India; ^2^Kaamadhenu Arts and Science College, Sathyamangalam 638503, India

## Abstract

There is increasing interest in natural food colorants like carotenoids and anthocyanins with functional properties. Red sorghum bran is known as a rich source for anthocyanins. The anthocyanin contents extracted from red sorghum bran were evaluated by biochemical analysis. Among the three solvent system used, the acidified methanol extract showed a highest anthocyanin content (4.7 mg/g of sorghum bran) followed by methanol (1.95 mg/g) and acetone (1 mg/g). Similarly, the highest total flavonoids (143 mg/g) and total phenolic contents (0.93 mg/g) were obtained in acidified methanol extracts than methanol and acetone extracts. To study the health benefits of anthocyanin from red sorghum bran, the total antioxidant activity was evaluated by biochemical and molecular methods. The highest antioxidant activity was observed in acidified methanol extracts of anthocyanin in dose-dependent manner. The antioxidant activity of the red sorghum bran was directly related to the total anthocyanin found in red sorghum bran.

## 1. Introduction

Anthocyanins are becoming increasingly important not only as food colorants but also as antioxidants. Anthocyanins are reported to have some therapeutic benefits including vasoprotective and anti-inflammatory properties [1] and anticancer [2] as well as hypoglycemic effects [3]. There is a rising demand for natural sources of food colorants with nutraceutical benefits [4], and alternative sources of natural anthocyanins are becoming more important. Special features of the sorghum crop are very important in the world's human diet, with over 300 million people dependent on it [5]. Sorghum is one of the major staple foods in Africa, Middle East, and Asia. It is drought resistant and is, therefore, an extremely important commodity that provides necessary food and feed for millions of people living in semiarid environment worldwide. Sorghums have high levels of anthocyanins and other phenols concentrated in their brans [6, 7]. These sorghum bran fractions are potentially useful ingredients in various functional food applications and were shown to produce desirable attributes (e.g., attractive natural colour) without adversely affecting other sensory properties of foods. These ingredients are bound to play a crucial role in food applications as a diversified functional food base. However, to ensure their economic potential, the sorghum bran anthocyanins must be extracted in an efficient manner in which their original form is preserved as much as possible.

Oxidation is essential to many living organisms for the production of energy necessary for biological processes. Oxygen-centered free radicals, also known as reactive oxygen species (ROS), including superoxide, hydrogen peroxide, hydroxyl (HO–), peroxyl (ROO–), and alkoxyl (RO–), are produced *in vivo* during oxidation [8]. ROS are not only strongly associated with lipid peroxidation, leading to food deterioration, but are also involved in development of a variety of diseases, including cellular aging, mutagenesis, carcinogenesis, coronary heart disease, diabetes, and neurodegeneration [9, 10]. Although almost all organisms possess antioxidant defense and repair systems to protect against oxidative damage, these systems are insufficient to prevent the damage entirely [11, 12].

Antioxidative properties of anthocyanins arise from their high reactivity as hydrogen or electron donors, from the ability of the polyphenol-derived radicals to stabilize and delocalize the unpaired electron, and from their ability to chelate transition metal ions (termination of the Fenton reaction) [13]. Thus, anthocyanins may play a role as antioxidants.

The objective of the current research is to evaluate the antioxidant properties of anthocyanins from red sorghum bran.

## 2. Experimental Procedures

### 2.1. Samples

The brans of *Sorghum bicolor *(L.) red sorghum were collected from farmer's field in Tamil Nadu, India, and was stored at −20°C in the dark. All samples were decorticated using a PRL dehuller to obtain bran. Bran yield was 15% for each sample. Bran samples were ground through a UDY mill (1 mm mesh) before extraction and analysis. All analyses were conducted in triplicate.

### 2.2. Sample Extraction

The anthocyanin from red sorghum bran was extracted by the method followed by Joseph et al. [14]. Two solvents, methanol and acidified methanol, were used for extraction procedure. For 0.5 g sample, 10 mL of solvent was added in 50 mL centrifuge tubes, and the samples were kept in an orbit shaker (Neolab) for 2 hrs at low speed. Samples were then stored at −20°C for over night in the dark to allow for maximum diffusion of phenolics from the cellular matrix. Samples were then kept at room temperature and centrifuged at 7,000 ×g for 10 min, taken for analysis. Residues were rinsed with two additional 10 mL volumes of solvent for 5 min and centrifuged at 7,000 ×g for 10 min, and taken for analysis. The three aliquots were mixed and stored at −20°C in the dark until further biochemical analysis.

### 2.3. Analytical Procedures

#### 2.3.1. Flavanoid Confirmation Test

The flavanoid confirmation test was determined by the method of Harbone 1998.

#### 2.3.2. Stability at Variable pH

The anthocyanin stability was tested by treating 1 mL of sample with 1 mL of pH 1.0 and 4.5 solutions. The color change was observed [15].

#### 2.3.3. Determination of Total Phenolic Content

Total phenolic contents of anthocyanin samples were determined by the Folin-Ciocalteu's method [16]. Briefly, aliquots of 0.1 g powder of anthocyanin samples were dissolved in 1 mL of deionised water. This solution (0.1 mL) was mixed with 2.8 mL of deionized water, 2 mL of 2% sodium carbonate (Na_2_CO_3_), and 0.1 mL of 50% Folin-Ciocalteu's reagent. After incubation at room temperature for 30 min, the absorbance of the reaction mixture was measured at 750 nm against a deionized water blank using a spectrophotometer (Geneysis 5). Gallic acid was chosen as a standard to get a seven-point standard curve (0–200 mg/L). The levels of total phenolic contents in sorghum bran were determined using the standards curve. The data obtained from sorghum bran were expressed as milligram of gallic acid equivalents (GAEs)/g powder and converted to milligram gallic acid equivalents (GAEs)/100 g of sorghum bran.

#### 2.3.4. Determination of Total Flavonoid Content

The total flavonoid content was quantified by using aluminum chloride colorimetric method described by Chang et al. [17, 18]. In brief, aliquots of 0.1 g of samples were dissolved in 1 mL of deionized water. This solution (0.5 mL) was mixed with 1.5 mL of 95% alcohol, 0.1 mL of 10% aluminium chloride hexahydrate (AlCl_3_), 0.1 mL of 1 M potassium acetate (CH_3_COOK), and 2.8 mL of deionised water. After incubation at room temperature for 40 min, the reaction mixture absorbance was analysed using spectrophotometer (Geneysis 5) at 415 nm. The deionized water was used as a blank. Quercetin was chosen as a standard to get a seven-point standard curve (0–50 mg/L). The levels of total flavonoid contents in sorghum bran were determined in triplicate, respectively. The data obtained from sorghum bran were expressed as milligram of quercetin equivalents (GAE)/g powder. The data were expressed as milligram equivalents (GAE)/g powder. Finally, the data were converted to milligram quercetin equivalents (GAE)/100 g of sorghum bran.

#### 2.3.5. Determination of Total Anthocyanin

The total amount of anthocyanin content was determined by using pH differential method. A spectrophotometer was used for the spectral measurements at 210 nm and 750 nm [19]. The absorbance of the samples (A) was calculated as follows:

(1) $$\begin{array}{cc} & \text{anthocyanin}\;\text{pigment}\;\text{content}\;\left(\text{mg}/\text{liter}\right)\\ & \;\;=\frac{\text{A}\times \text{MW}\times \text{DF}\times 1000}{\varepsilon \times 1}, \end{array}$$

where A = (Absorbance *λ* vis-max-A750) at pH 1.0–(Absorbance *λ* vis-max-A750) at pH 4.5 molecular weight of anthocyanin (cyd-3-glu) = 449, extraction coefficient (*ε*) = 29,600, and DF = diluted factor. 

### 2.4. Antioxidant Assays

#### 2.4.1. Scavenging Activity of DPPH Radical

Scavenging activity of anthocyanins against DPPH radicals was assessed according to the method of Larrauri et al. [20] with some modifications. Briefly, 0.1 mL of various concentrations of sorghum anthocyanins (1, 10, 50, 100 *μ*g/mL) was mixed with 2.9 mL of 0.1 mM DPPH-methanol solution. Mixed solutions were incubated for 30 min at 25°C in dark; the decrease in the absorbance at 517 nm was measured. Methanol was used as control instead of antioxidant solution while blanks contained methanol instead of DPPH solution. In the experiment, L-ascorbic acid and BHT were used as standards. The inhibition of DPPH radicals by the samples was calculated according to the following equation:

(2) $$\begin{array}{cc} & \text{DPPH-scavenging}\;\text{activity}\;\left(\text{\%}\right)\\ & =\left[1-\frac{\left(\text{absorbance}\;\text{of}\;\text{the}\;\text{sample}-\text{absorbance}\;\text{of}\;\text{blank}\right)}{\text{absorbance}\;\text{of}\;\text{the}\;\text{control}}\right]\\ & \;\times 100. \end{array}$$

#### 2.4.2. Hydroxyl Radical Scavenging Activity

The hydroxyl radical scavenging activity was determined based on the protocol described by Singh et al. [21]. of various concentrations of sorghum anthocyanins (1, 10, 50, 100 *μ*g/mL) was taken in different test tubes. 1.0 mL of iron-EDTA solution (0.1% ferrous ammonium sulfate and 0.26% EDTA), 0.5 mL of DMSO (0.85% v/v in 0.1 M phosphate buffer, pH 7.4) were added to these tubes, and the reaction was initiated by adding 0.5 mL of 0.22% ascorbic acid. Test tubes were capped tightly and heated on a water bath at 80–90°C for 15 min. The reaction was terminated by the addition of 1 mL of ice-cold TCA (17.5%w/v). 3 mL of the Nash reagent (75 g of ammonium acetate, 3 mL of glacial acetic acid, and 2 mL of acetyl acetone were mixed and raised to 1 L with distilled water) was added to all of the tubes and left at room temperature for 15 min for the color development. The intensity of the yellow color formed was measured spectrophotometrically at 412 nm against the reagent blank. L-ascorbic acid and butylhydroxytoluene (BHT) were used as standards. The percentage of hydroxyl radical scavenging activity was calculated by using the formula:

(3) $$\begin{array}{cc} & \text{\%}\;\text{of}\;\text{hydroxyl}\;\text{radical}\;\text{scavenging}\;\text{activity}\\ & \;\;=1-\frac{\text{absorbance}\;\text{of}\;\text{sample}}{\text{absorbance}\;\text{of}\;\text{blank}}\times 100. \end{array}$$

#### 2.4.3. Determination of Reducing Power

The reducing power was determined based on the method of Oyaizu [22]. A 0.25 mL aliquot of various concentrations of sorghum anthocyanins (1, 10, 50, 100 *μ*g/mL) was mixed with 2.5 mL of 200 mM sodium phosphate buffer (pH 6.6) and 2.5 mL of 1% potassium ferricyanide. The mixture was then incubated at 50°C for 20 min. After incubation, 2.5 mL of 10% trichloroacetic acid (w/v) were added; the mixture was centrifuged at 650 ×g for 10 min. About 5 mL aliquot of the upper layer was mixed with 5 mL of distilled water and 1 mL of 0.1% ferric chloride, and the absorbance of the mixture was measured at 700 nm. L-ascorbic acid and butylhydroxytoluene (BHT) were used as standards. A higher absorbance indicated a higher reducing power.

#### 2.4.4. Determination of Superoxide Radical-Scavenging Activity

Superoxide radicals were generated by the modified protocol described by Siddhurajuna et al. [23] all the solutions were prepared using 0.05 M phosphate buffer (pH 7.8). The photoinduced reactions were performed in aluminium foil-lined box with two 30 W fluorescent lamps. The distance between the reaction solution and the lamp was adjusted until the intensity of illumination reached about 4000 lux. A 30 *μ*L aliquot of various concentrations of sorghum anthocycanins (1, 10, 50, 100 *μ*g/mL) was mixed with 3 mL of reaction buffer solution (1.3 mm riboflavin, 13 mM methionine, 63 *μ*M nitro blue tetrazolium and 100 *μ*M EDTA, pH 7.8). The reaction solution was illuminated for 15 min at 25°C. The reaction mixture, without sample, was used as a control. L-ascorbic acid and butylhydroxytoluene (BHT) were used as standards. The scavenging activity was calculated as follows:

(4) $$\begin{array}{cc} & \text{scavenging}\;\text{activity}\;\left(\text{\%}\right)\\ & \;\;=\left(1-\frac{\text{absorbance}\;\text{of}\;\text{the}\;\text{sample}}{\text{absorbance}}\right)\times 100. \end{array}$$

#### 2.4.5. Metal Chelating Activity

The chelation of ferrous ions by the extract was estimated by the method of Dinis et al. [24] with slight modification and compared it with EDTA, BHT, and that of ascorbic acid. The chelation test initially includes the addition of ferrous chloride. The antioxidants present in the samples chelate the ferrous ions from the ferrous chloride. The remaining ferrous combine with ferrozine to form ferrous-ferrozine complex. The intensity of the ferrous-ferrozine complex formation depends on the chelating capacity of the sample, and the colour formation was measured at 562 nm (Shimadzu UV-Vis 2450). Different concentrations of standard and sorghum anthocycanins (1, 10, 50, 100 *μ*g/mL) were added to a solution of 100 *μ*L FeCl_2_ (1 mM). The reaction was initiated by the addition of 250 *μ*L ferrozine (1 mM). The mixture was finally quantified to 1.3 mL with methanol, shaken vigorously and left at room temperature for 10 min., after the mixture had reached equilibrium, the absorbance of the solution was measured spectrophotometrically at 562 nm. All the test and analysis were done in duplicates, and average values were taken. L-ascorbic acid and butylhydroxytoluene (BHT) were used as positive control. The percentage inhibition of ferrous ferrozine complex formation was calculated using the formula: % = 1 − As/Ac × 100, where “Ac” is the absorbance of the control and “As” is the absorbance of the sample.

#### 2.4.6. Estimation of Anti-FeCl_2_-H_2_O_2_-Stimulated Linoleic Acid Peroxidation

The effect of anti-FeCl_2_-H_2_O_2_-stimulated linoleic acid peroxidation was determined by the method as described by Duh [25]. In brief, 0.2 mL of various concentration sorghum anthocycanins (1, 10, 50, 100 *μ*g/mL) were added to a solution of 0.1 M linoleic acid (0.2 mL), 2.0 mM FeCl_2_. H_2_O (0.2 mL), and 0.2 M phosphate buffer (pH 7.4, 5 mL). The reaction mixture was incubated at 370°C for 24 h. After incubation, 0.2 mL of BHA (20 mg/mL), 1 mL of thiobarbituric acid (TBA)(1%), and 1 mL of trichloro acetic acid (TCA)(10%) were added to the mixture, which was heated for 30 min in a boiling water bath. After cooling, 5 mL of chloroform was added, and the mixture was centrifuged at 1000 ×g to give a supernatant. Absorbance of the supernatant was measured using spectrophotometer at 532 nm. 

#### 2.4.7. Determination of the Inhibitory Effect on Deoxyribose Degradation (Molecular Antioxidant Analysis)

Inhibitory effect of the anthocyanins on deoxyribose degradation was determined by measuring the reaction activity between either antioxidants or hydroxyl radicals (referred to as non-site-specific scavenging assay) or antioxidants and iron ions (referred to as site-specific scavenging assay) described by Lee et al. [26]. For non-site-specific scavenging assay, a 0.1 mL aliquot of different concentration of anthocyanin was mixed with 1 mL of reaction buffer (100 *μ*M FeCl_3_, 104 *μ*M EDTA, 1.5 mM H_2_O_2_, 2.5 mM deoxyribose, and 100 *μ*M L-ascorbic acid, pH 7.4) and incubated for 1 h at 37°C. A 1 mL aliquot of 0.5% 2-thiobarbituric acid in 0.025 M NaOH and 1 mL of 2.8% trichloroacetic acid were added to the mixture, and it was heated for 30 min at 80°C. The mixture was cooled on ice, and the absorbance was measured at 532 nm. Site-specific scavenging activity, which represented the ability of anthocyanins to chelate iron ions and interfere with hydroxyl radical generation, was measured using the same reaction buffer without EDTA. Percent inhibition of deoxyribose degradation was calculated as (1 − absorbance of sample/absorbance of control) × 100. Control—without sample.

#### 2.4.8. DNA Nicking Assay

DNA nicking assay was performed using genomic DNA by the method of Lee et al. [26]. A mixture of 10 *μ*L of sorghum anthocyanins (1 *μ*g/mL) and genomic DNA was incubated for 10 min at room temperature followed by the addition of 10 *μ*L of Fenton's reagent (30 mM H_2_O_2_, 50 *μ*M ascorbic acid, and 80 *μ*M FeCl_3_). The final volume of the mixture was made up to 20 *μ*L and incubated for 30 min at 37°C. The DNA was analysed on 1% agarose gel using ethidium bromide staining.

## 3. Results and Discussion

### 3.1. Anthocyanin Extraction

The total anthocyanin was extracted by 70% aqueous acetone, methanol, and acidified methanol as solvent system. Acidified methanol resulted in significantly higher values for the total anthocyanins than methanol and aqueous acetone (Table 1). In our results the anthocyanins extracted by aqueous acetone was much lower than methanol and acidified methanol. However, Lu and Foo [27] observed significant anthocyanin interaction with aqueous acetone to form pyranoanthocyanidins which significantly lowered quantities of detectable anthocyanins. This reaction was significantly affected by time of anthocyanin-acetone interaction and temperature. Our results did not observe such anthocyanin—solvent reactions in methanol and acidified methanol— and subsequently acidified methanol was proposed as a better solvent than methanol and aqueous acetone.

### 3.2. Flavonoid Confirmation Test

In the presence of FeCl_3_, the methanol and acidified methanol extracts showed brown color which confirmed the presence of Flavanoids [28]. In the presence of AlCl_3_ dark color was observed in methanol and acidified methanol extracts.

### 3.3. Stability at Variable pH

The sample appears red color at pH 1 and color disappeared at pH 4.5. The results were found to be same in the extracts of methanol and acidified methanol. Giusti and Wrolstad [29] reported that the anthocyanins are stable in low pH.

### 3.4. Total Phenolic Content

A wide variation in total phenolic content (TPC) was observed in three different solvent extracts from red sorghum bran (Table 1). The highest TPC was observed in acidified methanol extracts, 0.93 mg/g bran, while in methanol 0.58 mg/g bran and in aqueous acetone 0.14 mg/g bran. Awika et al. [30] reported that the highest concentrations of phenols were present in sorghum bran when acidified methanol was used as a solvent. It was also reported that the white sorghum bran fractions had phenol levels much lower than those measured in the black and brown sorghum brans. The pigmented sorghum varieties are a superior source of these beneficial compounds. Duodu et al. [31] reported that the chemical composition of any extract is dependent on the solvent used for extraction. Their results showed that acidified methanol extracts can preserve wider range of compounds, mainly phenolic compounds than aqueous extracts of sorghum bran.

### 3.5. Evaluation of Total Flavonoids

**Figure 1:**
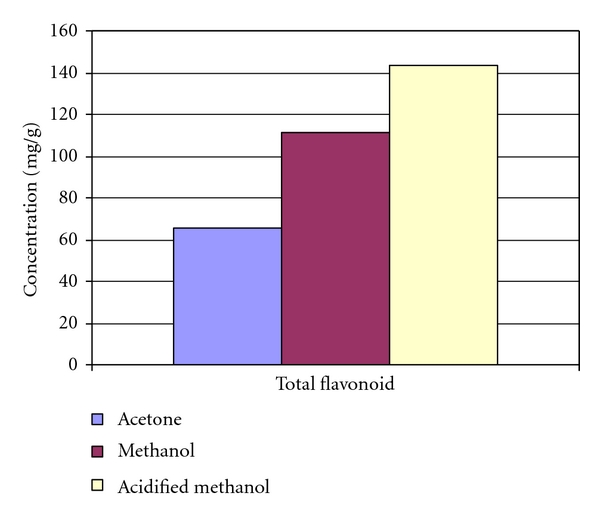
Flavonoid content of red sorghum bran in three different solvent extracts.

The anthocyanins are the major class of flavonoids in sorghum. The flavonoid content ranges from 65 to 142 mg/g bran in red sorghum. (Figure 1). Total flavonoid content in acidified methanol was 143 mg/g bran extract, while, for other extracts, total flavonoid content range was 65–111 mg/g bran of aqueous acetone and methanol extracts. Wharton et al. [32] reported that red pericarp sorghum has flavonol compounds, such as luteoforol and apiforol, which are produced from flavanones (i.e., naringenin and eriodictyol) and may be precursors of anthocyanidins in sorghum. A positive correlation between total phenols and flavanols (Table 1) suggests that total phenols are contributed mostly by flavanols in red pericarp sorghum. Similar results were reported by Linda Dykes et al. [33].

### 3.6. Anthocyanin Content

Acidified methanol resulted in significantly higher values for the mono- and total anthocyanins than methanol and aqueous acetone (Table 1). The total anthocyanins extracted by acidified methanol extracts were on average 59% higher than aqueous acetone extracts and 28% higher than methanol extracts. Several authors reported that aqueous acetone was better than various alcoholic solvents for extraction of fruit procyanidins, anthocyanins, and other phenols [34, 35]. However, since acidified methanol preservers better the extracted anthocyanins in their original form, it can be the solvent of choice for quantification and analysis of anthocyanins.

### 3.7. Antioxidant Analysis

#### 3.7.1. DPPH Radical Scavenging Activity

**Figure 2:**
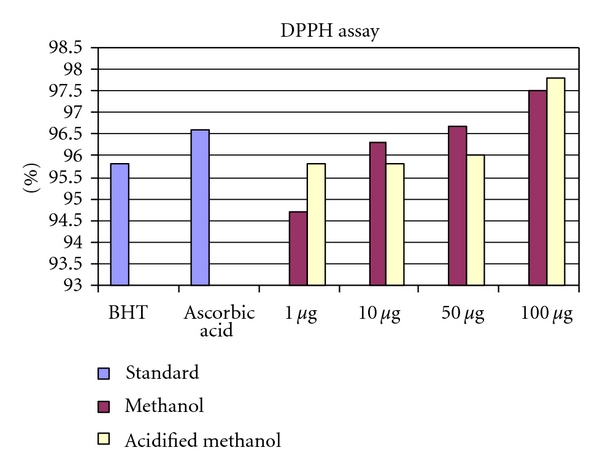
Concentration-dependent free radical scavenging activity of methanol and methanol extracts of anthocyanin from red sorghum bran.

Free radical scavenging is one of the known mechanisms by which antioxidants inhibit lipid peroxidation [8, 13]. The DPPH radical scavenging activity has been extensively used for screening antioxidants from fruits and vegetable juices or extract [36, 37]. DPPH radical scavenging activity of red sorghum bran anthocyanin, ascorbic acid, and BHT was shown in Table 2. The anthocyanin significantly inhibited the activity of DPPH radical in a dose-dependant manner. Anthocyanin had the highest scavenging activity followed by ascorbic acid and BHT. Antioxidant activities of extracted samples from sorghum bran using acidified methanol and methanol were compared (Figure 2). Samples extracted in acidified methanol had significantly higher antioxidant activity than those extracted in methanol. This implies that the acidified methanol is a more powerful solvent than methanol in extracting red sorghum antioxidants. At 1 *μ*g/mL, the scavenging effects were 94.7% and 95.8% for anthocyanins extracted from methanol and acidified methanol, respectively, while almost complete inhibition of DPPH radical activity was observed when the anthocyanins were used at 100 *μ*g/mL. It appears that red sorghum bran anthocyanins have a strong donating capacity and can efficiently scavenge DPPH radicals. Einbond et al. [38] reported that the DPPH radical scavenging activity of Surinam cherry, Jamaica cherry, and salal, Juboticaba, were due to the presence of large amount of anthocyanins. Awika et al. reported that 3-deoxy anthocyanins found in black sorghum had antioxidant activities that were similar to the anthocyanins found in the fruits and vegetables.

#### 3.7.2. Hydroxyl Radical Scavenging Activity

The sorghum anthocyanins exhibited the highest activity of 99% at 100 *μ*g/mL where as 73% inhibition was noted at 1 *μ*g/mL, respectively. Table 1 shows hydroxyl radical scavenging activity of anthocyanin extracts which increases with increasing concentration. The hydroxyl radical is an extremely reactive free radical formed in biological system and has been implicated as a highly damaging species in free radical pathology capable of damaging almost every molecule found in living cells. This species is considered to be one of the quick initiators of the lipid peroxidation process, abstracting hydrogen atoms from unsaturated fatty acids [39]. The acidified methanol extracts exhibited a highest activity of 99% at 100 *μ*g/mL than methanol extracts of 98.8% at 100 *μ*g/mL. This is similar to observations of several others who have reported a dose-dependent activity in sesame coat, pomegranate peel and seeds and grape pomace [17, 18, 21, 40]. The ability of ethanol extracts of leafy vegetables to quench hydroxyl radicals seems to be directly related to prevent ion in propagation of the process of lipid peroxidation. Hence, the extract seems to be a good scavenger of active oxygen species, thus reducing rate of chain reaction. A high positive correlation was observed between the polyphenol content and the hydroxyl radical scavenging activity. Shyamala et al. [41] reported that the leafy vegetables have a proton radical scavenging action, which is an important mechanism of antioxidants.

#### 3.7.3. Reducing Power

It has been reported that reducing power is associated with antioxidant activity and may serve as significant reflection on the antioxidant activity [17, 18, 42]. As shown in the Table 2, anthocyanins from red sorghum bran exhibited a higher reducing power than BHT and ascorbic acid, suggesting that it has strong electron-donating capacity. The reducing power of red sorghum bran anthocyanins at 100 *μ*g/mL ascorbic acid and BHT were 0.984, 0.577, and 0.138, respectively. Furthermore, a linear relationship was observed between concentration and reducing power of sorghum anthocyanins. The results indicated that the antioxidant activities of samples extracted in acidified methanol had significantly higher reducing power than those extracted in methanol. Earlier authors [25, 43, 44] have observed a direct correlation between antioxidant activities of certain plant extracts. The reducing properties are generally associated with the presence of reductones [25], which have been shown to exert antioxidant action by breaking the free radical chain by donating a hydrogen atom [45]. Reductones are also reported to react with certain precursors of peroxide, thus preventing peroxide formation.

#### 3.7.4. Superoxide Anion Scavenging Activity

**Figure 3:**
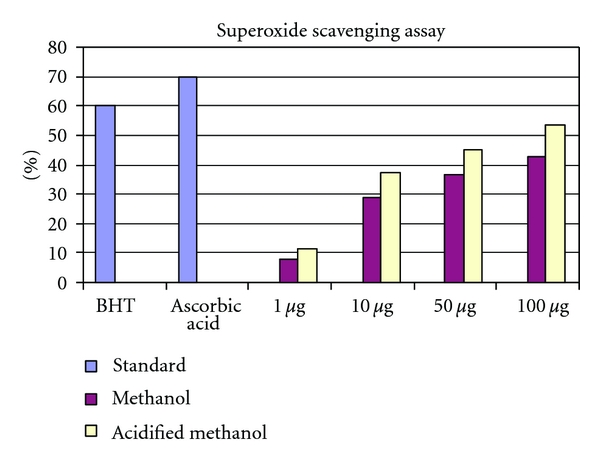
Concentration-dependent free radical scavenging activity of methanol and methanol extracts of anthocyanin from red sorghum bran.

The relative scavenging effects of red sorghum bran anthocyanin on superoxide radical are shown in Table 2. The anthocyanin extracts exhibited 71.92% scavenging acitivity at 100 *μ*g per mL.  Figure 3 shows the result of superoxide radical anion scavenging acitivity of anthocyanin extract, BHT, and ascorbic acid. Superoxide anion radicals are produced by a number of cellular reactions, including various enzyme system such as lipoxygenase, peroxidase, NADPH oxidase, and xanthine oxidase. Superoxide anion plays an important role in plant tissue and also involved in formations of other cell-damaging free radicals [8]. In the present study superoxide radical was generated by illuminating a solution containing riboflavin. Although superoxide is a relatively weak oxidant, it decomposes to form stronger reactive oxidative species, such as singlet oxygen and hydroxyl radicals, which initiate peroxidation of lipids [46]. In the present study, anthocyanin extracted from red sorghum bran effectively scavenged superoxide in a concentration-dependent manner. Further, superoxides are also known to indirectly initiate lipid peroxidation as a result of hydrogen peroxide formation, creating precursors of hydroxyl radicals [47]. These results clearly suggested that the antioxidant activity of anthocyanin is also related to its ability to scavenge superoxides.

#### 3.7.5. Metal Chelating Activity

The sorghum anthocyanins exhibited the highest activity of 85.2% at 100 *μ*g/mL whereas 48.3% inhibition was noted at 1 *μ*g/mL, respectively. The percentage inhibition values ranged from 46% to 85.2% (Table 2). The acidified methanol extracts had the highest chelating capacity than methanol, BHT, and ascorbic acid. The samples had better chelating capacity than standards based on percentage inhibiction values in terms of *μ*g/mL. Correlation was found between iron-chelating capacity and phenolic content. 

The ability of antioxidant to form insoluble metal complexes with ferrous ion or to generate steric hindrance that prevent interation between metal and lipid is evaluated using the metal ion chelating capacity assay [48]. Acitivity is measured by monitoring the decrease in absorbance of the red Fe^2+^ ferrozine complex as antioxidants compete with ferrozine in chelating ferrous ion [49].

#### 3.7.6. Anti-Ferric Chloride Hydrogen Peroxide System

The effect of anthocyanin extracts of red sorghum bran on the formation of malonaldehyde (MDA) from linoleic acid is show in Table 2. As the concentration of the antioxidant extracts increased, the formation of MDA decreased. A dose-dependent MDA inhibition in linoleic acid oxidation was evident. Methanol extracts showed a higher inhibition of MDA ranging from 73% to 92% at 100 *μ*g/mL than BHT and ascorbic acid. Iron salts are thought to react with H_2_O_2_ called the Fenton reaction, to make hydrogen radicals which bring about peroxide reaction of lipids [25].

#### 3.7.7. Hydrogen Peroxide Scavenging Activity

Hydrogen peroxide scavenging activities of the anthocyanin extracts, BHT, and ascorbic acid were measured at 230 nm. Hydrogen peroxide scavenging activities of the anthocyanin extracted by using acidified methanol were 47.9% at 1 *μ*g/mL, 88.6% at 10 *μ*g/mL, 90.4% at 50 *μ*g/mL, and 91.3% at 100 *μ*L/mL, respectively. Similarly hydrogen peroxide scavenging activities extracted using methanol were 40.6% at 1 *μ*g/mL, 69.5% at 10 *μ*g/mL, 85.3% at 50 *μ*g/mL and 90.2% at 100 *μ*L/mL. Table 2 shows, BHT (94.6%) had higher hydrogen peroxide scavenging activity than anthocyanin extracts. Park et al. [50] reported that the anthocyanin extracted from Black rice (*Heugjinjubyeo*) had good hydrogen peroxide scavenging activities in bran. 

#### 3.7.8. Inhibitory Effects of Deoxyribose Degradation (Molecular Antioxidant Analysis) 

Hydroxyl radical can be formed by the Fenton reaction in the presence of reduced transition metals such as Fe^2+^, and H_2_O_2_, which is known to be the most reactive of all the reduced forms of dioxygen and is thought to initiate cell damage *in vivo *[12, 51]. To determine whether sorghum anthocyanins reduce hydroxyl radical generation by chelating metal ions or by directly scavenging hydroxyl radicals, the effects of the anthocyanins or hydroxyl radical generated by Fe^3+^ ions were analyzed by determining the degree of deoxyribose degradation. Table 2 shows the concentration-dependent inhibition of hydroxyl radical induced deoxy ribose degradation by anthocyanins in both the site-specific and non-site-specific assays using the same concentration, relatively greater antioxidant activity was observed in the site-specific assay than non-site-specific assay, implying that the anthocyanins inhibited deoxyribose degradation mainly by chelating metal ions rather than by scavenging hydroxyl radical directly. In this system, anthocyanin extracts exhibited a stronger concentration-dependent inhibition of deoxyribose oxidation. Smith et al. [52] earlierly reported that molecules that can inhibit deoxyribose degradation are those that can chelate iron ions and render them in active or poorly active in a Fenton reaction. In the present study, in site specific system, we have demonstrated the iron chelating ability of the anthocyanin extracts. It is likely that the chelating effect of red sorghum bran anthocyanins on metal ions may be responsible for the inhibition of deoxyribose oxidation. Iron, a transition metal, is capable of generating free radicals from peroxides by the Fenton reaction and is implicated in many diseases [53]. Fe^2+^ has also been shown to produce oxyradicals, and lipid peroxidation and reduction of Fe^2+^ concentration in the Fenton reaction would protect against oxidative damage. Similar results were reported for extracts of *Opuntia ficus-indica *var. *saboten* [26] and *Hypericum perforatum L*. [54].

#### 3.7.9. DNA Nicking Assay

**Figure 4:**
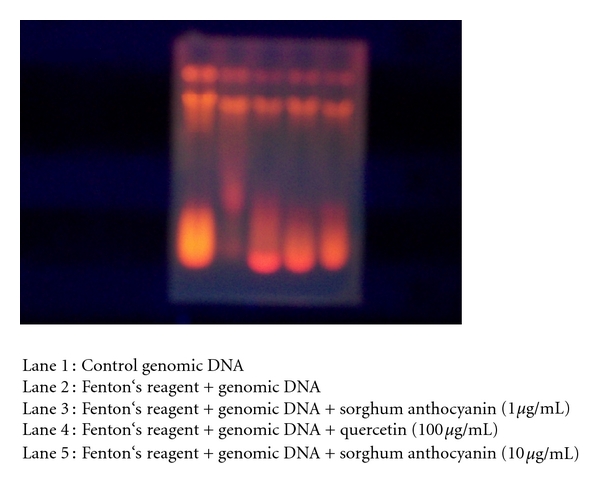
DNA nicking assay.

Hydroxyl radicals generated by the Fenton reaction are known to cause oxidatively induced breaks in DNA strands to yield its fragmented forms. The free radical scavenging effect of sorghum anthocyanins were studied on Genomic DNA damage. The anthocyanins showed significant reduction in the formation of nicked DNA and increased native form of DNA. Quercetin effectively protected DNA strand scission from *tert*-butyl hydroperoxide [55]. In biological systems metal binding can occur on DNA leading to partial site-specificity hydroxyl radical formation. Anthocyanins are potential protecting agents against the lethal effects of oxidative stress and offer protection of DNA by chelating redox-active transition metal ions. Mas et al. [56] suggested that anthocyanins have the ability to stabilize DNA triple-helical complex. So far no reports are available on protecting DNA damage of red sorghum anthocyanins (Figure 4). 

## 4. Conclusion

From the results it can be concluded that red sorghum bran has high amount of anthocyanins content on its bran. Reactive oxygen species plays a crucial role in a wide range of common diseases and age-related degenerative conditions including cardiovascular diseases, inflammatory conditions, and neurodegenerative diseases such as Alzheimer's disease, mutations and cancer [57]. So antioxidant capacity is widely used as a parameter to characterize food or medicinal plant, and their bioactive components. In this study, the antioxidant activity of the anthocyanins extracted from sorghum bran was evaluated, and it showed very strong antioxidant activity.

Thus, these results suggest that anthocyanin extracted from red sorghum bran can be used as antioxidant material and food addictives.

## Figures and Tables

**Table 1:** Phenolic composition, flavonoid and anthocyanin content of red sorghum bran in three different solvent extracts.

Red sorghum bran	Total phenols (mg gallic acid equiv/g)	Total flavonoids (mg queractin equiv/g)	Anthocyanins (mg cyanidin 3-glucoside equiv/g) (mg/g)
Acetone extract	0.14 ± 0.03	65 ± 0.19	1.00 ± 0.11
Methanol extract	0.58 ± 0.04	111 ± 0.20	1.95 ± 0.13
Acidified methanol extract	0.93 ± 0.06	143 ± 0.23	4.7 ± 0.20

Values are mean (*n* = 3) ± SD (*n* = 3, *P* < 0.05).

**Table 2:** Concentration dependent free radical scavenging activity of methanol and methanol extracts of anthocyanin from red sorghum bran.

Antioxidant assays	Standards (%)		Methanol extract (%)				Acidified methanol extract (%)			
	BHT	Ascorbic acid	1 *μ*g	10 *μ*g	50 *μ*g	100 *μ*g	1 *μ*g	10 *μ*g	50 *μ*g	100 *μ*g
DPPH assay	95.8	96.6	94.7	96.3	96.7	97.5	95.8	95.8	96	97.8
Superoxide radical scavenging assay	60.4	69.9	7.6	29	36.5	42.5	11.2	37.5	45	53.8
Hydroxyl radical scavenging assay	99.8	69.9	30.4	84.5	98.5	98.8	73	89.7	98.6	99
Reducing power (Absorbance)	0.138	0.577	0.024	0.184	0.552	0.840	0.014	0.037	0.445	0.984
Metal chelating	79	83	46	74.4	80.3	85.1	48.3	75.2	83.7	85.2
Hydrogen peroxide	94.6	—	40.6	69.5	85.3	90.2	47.9	88.6	90.4	91.3
Deoxyribose degradation site-specific assay	99.1	48.4	85	89.6	91.3	91.8	72	88.9	91.7	91.8
Deoxyribose degradation non-site-specific assay	80.2	33.9	74.8	79.8	81.9	86.6	59.8	79	81.4	82
Anti FeCl_3_–H_2_O_2_ stimulated Linoleic acid	77.2	87.8	82.2	83.5	85.5	86.2	73.1	81	84.3	92
